# The Relationship Between the Dark Triad and Internet Adaptation Among Adolescents in China: Internet Use Preference as a Mediator

**DOI:** 10.3389/fpsyg.2019.02023

**Published:** 2019-08-30

**Authors:** Can Can Jin, Bo Chen Wang, Ai Tong Ji

**Affiliations:** Department of Psychology, School of Humanities and Social Sciences, Beijing Forestry University, Beijing, China

**Keywords:** Dark Triad, Machiavellianism, psychopathy, narcissism, internet use preference, Internet adaptation, Chinese adolescent

## Abstract

This study aims to examine the relationship among the Dark Triad personality traits, Internet use preferences and Internet adaptation. In all, 1927 middle school students (aged 11–18) from Beijing and Kunming in China completed a self-reported questionnaire that measured the Dark Triad, Internet use preferences (information communication, recreation, information acquisition and online transaction) and Internet adaptation (positive/negative Internet adaptation). Correlations revealed that there were significant correlations among the Dark Triad, Internet use preferences and Internet adaptation. Multiple regression analysis indicated that psychopathy predicted Internet adaptation more significantly than Machiavellianism or narcissism. Information acquisition positively predicted positive Internet adaptation and negatively predicted negative Internet adaptation, and recreation and online transaction were just the opposite. The SEM results suggested that Internet use preferences partially mediated the relationship between the Dark Triad and Internet adaptation. We discussed the importance of the combined effect of the Dark Triad and Internet use preferences on Internet adaptation.

## Introduction

Recently, more and more Chinese adolescents use the Internet to study, communicate and shop. Until December 2018, there have been approximately 145 million Internet users aged 10–19 in China (China Internet Network Information Center, 2019). The issue of adolescents’ Internet use has concerned researchers and parents. The relationship between an individual and the Internet, called Internet adaptation, is one of the most important factors in Internet use. Internet adaptation is a special kind of social adaptation (Yang and Jin, 2007). According to Ji (2018), Internet adaptation is defined as a “harmonious relationship among individual, network and reality.” It is very useful for the development of adolescents’ mental and physical health (Ji, 2018). There are two basic theoretical frameworks of social adaptation: “function-structure” theoretical perspectives and “process-structure” theoretical perspectives. Adaptation is regarded as a final outcome in the former, which can be divided into positive adaptation and negative adaptation. Adaptation is regarded as a process from start to finish in the latter (Chen, 2001; Zou et al., 2012).

Similarly, theoretical perspectives of social adaptation can be used for reference to discuss Internet adaptation. Considering possible measurement and practical applications, we used the “function – structure” perspectives to discuss Internet adaptation. On the one hand, from the perspective of “function,” positive Internet adaptation means that individuals can balance the relationship between themselves and the Internet environment, and use Internet reasonably to facilitate their development. Negative Internet adaptation refers to individuals cannot coordinate the relationship between themselves and the Internet environment well, develop Internet deviant or antisocial behaviors which impede individual growth. On the other hand, from the perspective of “structure,” according to critical development task theory (Masten et al., 1995), interpersonal relationship, academic achievement and normative behavior are the main development tasks from childhood through adolescence, which are also the main performance aspects of individuals in the Internet environment. Based on this, negative Internet adaptation is mainly manifested in Internet Interpersonal Orientation (e.g., ignoring real interpersonal relationships and being addicted to social networking) (Hughes et al., 2012), Academic Escape (e.g., surfing the Internet to escape from schoolwork) (Liu and Jin, 2018) and Internet problem behaviors (e.g., cyberbullying and browsing pornographic and violent websites) (Kowalski et al., 2014; Zhou and Liu, 2016). Positive Internet adaptation is mainly manifested in Internet relational use, which includes complying with network norms, rational expression on the Internet, and rejecting negative Internet information (Hughes et al., 2012; Lee, 2014).

Internet adaptation is influenced by many factors, among which Internet use preferences were significant predictors of Internet adaptation. Internet use preferences mainly refers to information communication (e.g., communicating via WeChat, social networking sites or e-mail), Internet recreation (e.g., online videos, music, games or literature), Internet information acquisition (e.g., Internet information search) and Internet transactions (e.g., online ordering takeaway or taxi booking). The rate of the above categories of Internet use ranged from 47.9 to 95.5% (China Internet Network Information Center, 2019). According to the uses and gratification theory (Katz et al., 1973; Song et al., 2004), these different Internet use preferences are caused by a variety of social and psychological needs, which include escapism, obtaining information and recreation. In turn, they lead users to participate in different kinds of media activities on the Internet, which meet the demands of various Internet users (Lei, 2016).

Previous studies have focused more on negative Internet adaptation caused by Internet use preferences (Song et al., 2004; Hughes et al., 2012; Kowalski et al., 2014). To some degree, preferences for recreation, playing games and making friends on the Internet might increase the possibility of Internet addiction or Internet deviant behaviors (Zhou and Liu, 2016). However, the positive effect of the Internet should not be ignored. For example, the emergence of the Internet has greatly reduced the cost of information acquisition. Individuals with information acquisition preferences were more likely to search for learning materials and use online learning platforms, and their online behaviors tend to be positive and appreciated. In a manner of speaking, whether Internet adaptation is positive or negative mainly depends on Internet use preferences. Therefore, hypothesis 1 was that information acquisition Internet use preference would be positively related to positive Internet adaptation and negatively related to negative Internet adaptation, whereas information communication, recreation and online transaction dimensions would be negatively related to positive Internet adaptation and positively related to negative Internet adaptation.

Personality is also a predictor of Internet adaptation. Much research has shown that personality traits, such as impulsivity, sensation-seeking, extraversion, neuroticism and psychoticism are closely related to Internet addiction among adolescents (Gosling et al., 2011; Deng et al., 2014; Yao et al., 2014; Kayis et al., 2016; He et al., 2017). Other evidence has indicated that nervousness from Eysenck’s personality significantly predicted a series of Internet deviant behaviors, such as extreme behaviors, pornographic behaviors, deceptive behaviors and aggressive behaviors (Luo et al., 2011). However, in general, researchers have paid more attention to the relationship between negative Internet adaptation and personality, ignoring the relationship between positive (reasonable) Internet behavior and personality.

Although traditional personality traits (e.g., Big Five personality traits) are widely accepted, they excessively focus on the “bright” aspects of personality and lack consideration for the “impolite” aspects of personality (Block, 2010). Recently, Dark Triad traits were developed to make up for the limitations of traditional personality research and have gradually been noticed in China (Stead et al., 2012; Qin and Xu, 2013; Zhang and Zhang, 2014). There is little doubt that the Dark Triad traits represent three related traits: Machiavellianism, psychopathy and narcissism. They appear to be linked by a core of disagreeableness, lacking humility, feeling they can predict future outcomes and a fast or opportunistic life strategy (Figueredo et al., 2006; Jonason and Tost, 2010; Jonason et al., 2011).

It should be noted that the Dark Triad is not a maladaptive individual trait but the result of an individual’s active adaptation to the environment (Qin and Xu, 2013). From this perspective, the Internet can be regarded as a specific environment of modern information society. Therefore, Dark Triad personality traits are likely to be related to Internet adaptation and behaviors. However, so far, the relationship between the Dark Triad traits and Internet adaptation has not been clearly studied. Limited evidence has shown that psychopathy and narcissism significantly predict cyberbullying (Ang et al., 2010; Carpenter, 2012; Pabian et al., 2015); Individuals with high narcissism were more likely to use social networking sites frequently (Eksi, 2012). Based on these findings, we offered hypothesis 2: that Dark Triad traits would negatively predict positive Internet adaptation and positively predict negative Internet adaptation.

Dark Triad traits are also linked to Internet use preferences. According to life history theory, individuals with the Dark Triad personality traits tend to attach more importance to reproduction than subsistence when considering how to allocate their resources. They are more likely to believe the living environment is highly unstable and unpredictable and they would like to adopt a fast or opportunistic life strategy, with a reduced ability to delay gratification and less self-control, and tend to love adventure and seizing the day (Figueredo et al., 2007; Zhang and Zhang, 2014). Based on the above statements, individuals with higher scores on Dark Triad traits are more likely to pursue Internet use that fulfills instant needs and short-term interests, such as recreation, leisure, and online transaction, and to keep away from Internet use that fulfills long-term interests and requires a lot of cognitive effort, such as online learning or searching for Internet information. Further evidence has revealed that Internet users with different personality traits develop different Internet use preferences based on user requirements and various services provided by the Internet. Long-term use preferences further have an impact on the outcome of Internet use, also known as Internet adaptation (Amichai-Hamburger, 2002; Lei, 2016). Therefore, hypothesis 3 was offered that Internet use preferences would mediate the relationship between the Dark Triad and Internet adaptation.

## Materials and Methods

### Participants

In all, 1927 middle school students (1,139 boys, 775 girls, 13 did not identify sex; 799 junior middle school students, 1118 senior middle school students, 10 did not identify middle school or high school) aged 11–18 (*M* = 15.07, *SD* = 1.95) were recruited from 4 middle schools from Beijing and Kuming in China. Independent sample *T*-tests were conducted to examine differences between students from Beijing and Kunming. The results indicated no significant differences in Internet adaptation (*t* = 0.54, *p* > 0.05), Internet use preferences (*t* = 1.93, *p* > 0.05) or Dark Triad traits (*t* = 1.83, *p* > 0.05) between students from Beijing and Kunming. All participants signed an informed consent form.

### Procedure

First, 451 middle school students from a middle school in Beijing participated in the pre-test to revise the scales and examine the reliability and validity of the scales.

Second, a total of 1,927 middle school students were recruited from 4 middle schools in Beijing and Kunming. After obtaining the informed consent signed by the parents and the head teacher of the adolescent participant, participants were asked to complete the Chinese–language self–report questionnaires, including Chinese Adolescent’s Internet Adaptation Scale, Internet use preference scale, Dark Dozen and SES items, during the normal school day. Self-reported questionnaires were handed out to collect the data in an organized classroom setting. The adolescents completed them during a single class period. We used SPSS 22.0 and AMOS 17.0 to analyze the data. The study procedures were conducted following approval by the Ethics Review Committee of Beijing Forestry University.

### Measures

#### Internet Adaptation Scale

Internet adaptation among adolescents was measured using Ji’s (2018) Chinese Adolescent’s Internet Adaptation Scale (CAIAS). The CAIAS has 27 items and measures four components of Internet adaptation: Internet Problem Behaviors (e.g., I have sent insulting or hurtful messages to my classmates or friends through the Internet), Internet Interpersonal Orientation (e.g., My online friends know me better than my real life friends), Academic Escape (e.g., I don’t have enough time to do my homework due to I spend too much time on the Internet) and Internet Relational Use (e.g., I communicate and express myself rationally on the Internet). The total score of negative Internet adaptation is equal to the total average score of the first three components, while the total score of positive Internet adaptation is equal to the average score of the last component. The respondents indicated how important each statement was for them on a 5-point Likert scale ranging from 1 (strongly disagree) to 5 (strongly agree). The overall alpha coefficient of the CAIAS was 0.82, with a range from 0.64 to 0.78 for the four subscales. The results indicated good fit for the measurement model: χ^2^*/df* = 6.61; CFI = 0.92; GFI = 0.96; TLI = 0.90; NFI = 0.91; RMSEA = 0.06.

#### Internet Use Preference Scale

The Internet use preference scale was developed from the original Internet Use Preference Scale for Mainland Chinese Adolescents (Cheng and Liu, 2010), with three dimensions of information communication, recreation and information acquisition. Information communication includes blogging, chatting on WeChat, uploading pictures and browsing friends’ cyberspace. Recreation refers to watching network videos, reading graphic novels and playing games. Information acquisition includes searching for information relevant to the course, learning computer technology, and querying life information (e.g., weather forecast, maps). Recently, online payment and online trading has become an important aspect of people’s network life, and online transaction accounted for 72.5% of total Internet use (China Internet Network Information Center, 2019). Therefore, online transaction was added in the revised Internet use preference scale, with items including “online shopping, online payment, online booking ticket and hotel online group-buying.” The scale was revised to 20 items after testing with 451 junior and high school students. Participants responded using a 4-point format ranging from 1 (never) to 4 (often). The overall alpha coefficient of the scale was 0.87, with a range from 0.68 to 0.83 for the four subscales. The results indicated a good fit for the measurement model: χ^2^/*df* = 10.22, CFI = 0.91, GFI = 0.93, TLI = 0.89, NFI = 0.90, RMSEA = 0.07.

#### Dark Triad Traits

The Dark Triad was measured using Geng et al. (2015) Revised Chinese Version of the “Dark Dozen”. The Dark Dozen is 12 items and measures Machiavellianism (4 items, e.g., “I tend to manipulate others to get my way”), psychopathy (4 items, e.g., “I tend to lack remorse”), and narcissism (4 items, e.g., “I tend to want others to admire me”). Participants were asked to indicate the degree to which each item applied to them using a 7-point response format ranging from 1 (Strongly disagree) to 7 (Strongly agree). The overall alpha coefficient of the Dark Dozen Scale was 0.86, with a range from 0.77 to 0.88 for the three subscales.

#### Self-Designed Demographic Questionnaire

The demographic questionnaire included gender, grade, parents’ occupation, parents’ education level, average monthly income and online hours per week.

According to Jin (2013) and Zhou et al. (2008), family socioeconomic status includes education level, parents’ occupational prestige, and average monthly household income. Paternal and maternal education level were coded into the following three levels: (a) junior middle school graduate or lower; (b) high school graduate, vocational school or technical school; and (c) college degree or higher. The education level for both parents was assigned a number ranging from 1 to 3, with higher numbers indicating greater educational attainment. The higher educational level of the father or mother was labeled the parents’ education level.

Similarly, paternal and maternal occupations were coded into three categories: (a) farming, forestry, animal husbandry and fishery workers, and laid-off workers; (b) middle occupational level, such as clerical workers in government offices, state enterprises, workers involved in commerce and the service industry, and self-employed workers; and (c) management, such as government officials, large or medium business executives, and senior technical personnel. The occupation ratings for both parents were assigned a number ranging from 1 to 3, with higher numbers indicating greater occupational status. The higher occupational level of the father or mother was labeled the parents’ occupational reputation.

The average monthly household income was also coded into three levels: (a) less than or equal to 4,000 RMB, (b) 4,000–10,000 RMB, and (c) more than 10,000 RMB. One dollar is equal to approximately six RMB. Household income was assigned a number from 1 to 3, with higher numbers indicating greater household income. We added the three indices and constructed a composite measurement of the overall objective family SES. The family SES scores ranged from 3 to 9.

The number of hours spent online per week was equal to the average number of hours online per day times the number of days online per week.

## Results

### Common Method Biases

Common method biases were examined using *post hoc* Harman’s one-factor analysis to check whether the variance of all included constructs could be largely attributed to a single factor (Podsakoff et al., 2003; Zhou and Long, 2004). The exploratory factor analysis revealed that three common factors with clear meanings were extracted from all included constructs, and 24.03% (<40%) of the total variance was attributed to the greatest factor, which indicated that this research was not pervasively affected by common method biases.

### Pearson Correlations Among the Dark Triad, Internet Use Preferences and Internet Adaptation

Table 1 shows the Pearson correlation analysis among the key study variables. Information acquisition was correlated negatively with Machiavellianism, psychopathy and negative Internet adaptation and was correlated positively with positive Internet adaptation. Recreation was correlated positively with the Dark Triad and negative Internet adaptation. Online transaction was correlated positively with Machiavellianism, narcissism and negative Internet adaptation. More remarkably, information communication was positively correlated with both positive Internet adaptation and negative Internet adaptation. The Dark Triad was positively correlated with negative Internet adaptation and negatively correlated with positive Internet adaptation (see Table 1). The results from Pearson correlations indicated that information acquisition is a positive dimension, whereas recreation and online transaction are negative dimensions. However, the nature of information communication was not clear. Moreover, family socioeconomic status (SES) and online hours per week were significantly correlated with positive and negative Internet adaptation, but they were not the key variables in this study and were excluded from further analyses, which means that the effect of SES and online hours on Internet adaptation needs to be controlled in future analyses.

**TABLE 1:** Correlations among sociodemographic variables, the Dark Triad, Internet use preferences and Internet adaptation.

	***M***	***SD***	**1**	**2**	**3**	**4**	**5**	**6**	**7**	**8**	**9**	**10**
1	6.45	2.15	–									
2	11.27	14.31	–0.10^∗∗∗^	–								
3	12.12	3.74	0.02	0.13^∗∗∗^	–							
4	18.28	3.84	0.01	0.18^∗∗∗^	0.47^∗∗∗^	–						
5	15.17	3.38	0.11^∗∗∗^	0.02	0.35^∗∗∗^	0.29^∗∗∗^	–					
6	9.80	3.57	–0.06^∗∗^	0.16^∗∗∗^	0.45^∗∗∗^	0.43^∗∗∗^	0.32^∗∗∗^	–				
7	1.91	1.31	0.05^∗^	0.08^∗∗∗^	0.01	0.11^∗∗∗^	–0.10^∗∗∗^	0.05^∗^	–			
8	2.04	1.23	−0.05^∗^	0.09^∗∗∗^	–0.02	0.09^∗∗∗^	–0.19^∗∗∗^	0.02	0.62^∗∗∗^	–		
9	3.61	1.70	0.06^∗^	0.09^∗∗∗^	0.11^∗∗∗^	0.17^∗∗∗^	0.01	0.10^∗∗∗^	0.33^∗∗∗^	0.28^∗∗∗^	−	
10	4.10	0.74	0.20^∗∗∗^	–0.09^∗∗∗^	0.06^∗∗^	–0.02	0.29^∗∗∗^	–0.02	–0.19^∗∗∗^	–0.27^∗∗∗^	−0.05^∗^	–
11	2.07	0.63	–0.18^∗∗∗^	0.24^∗∗∗^	0.17^∗∗∗^	0.31^∗∗∗^	–0.10^∗∗∗^	0.25^∗∗∗^	0.38^∗∗∗^	0.41^∗∗∗^	0.24^∗∗∗^	–0.38^∗∗∗^

*^∗^*p* < 0.05 ^∗∗^*p* < 0.01 ^∗∗∗^*p* < 0.001. (1) Family socioeconomic status (2) Online hours per week (3) Information communication (4) Recreation (5) Information acquisition (6) Online transaction (7) Machiavellianism (8) Psychopathy (9) Narcissism (10) Positive Internet adaptation (11) Negative Internet adaptation.*

### Regression Analysis of the Effects of the Dark Triad and Internet Use Preference on Internet Adaptation

Table 2 shows the results of the hierarchical multiple regression analysis for the effects of the Dark Triad and Internet use preference on the two kinds of Internet adaptation. The results showed that when controlling for family SES and online hours per week, Positive Internet adaptation was predicted negatively by Machiavellianism, psychopathy, recreation and online transaction and was predicted positively by information acquisition. Negative Internet adaptation was predicted positively by the Dark Triad, information communication, recreation and online transaction and was predicted negatively by information acquisition. Compared to other dimensions of the Dark Triad, psychopathy was a stronger predictor of both positive Internet adaptation (β = −0.16, *p* < 0.001) and negative Internet adaptation (β = 0.21, *p* < 0.001). Compared to other dimensions of Internet use preferences, information acquisition was a stronger predictor of positive Internet adaptation (β = 0.27, *p* < 0.001), and recreation was a stronger predictor of negative Internet adaptation (β = 0.21, *p* < 0.001) (see Table 2).

**TABLE 2:** Hierarchical multiple regression analysis of the Dark Triad and Internet use preferences predicting Internet adaptation.

	**Positive Internet adaptation**				**Negative Internet adaptation**			
		
**Independent variable**	**β_m__odel__1_**	**β_model__2_**	**Δ*R*^2^/Δ*F***	***R*^2^/*F***	**β_m__odel__1_**	**β_model__2_**	**Δ*R*^2^/Δ*F***	***R*^2^/*F***
1. *Sociodemographic*^a^			0.04/45.05^∗∗∗^	0.04/45.05^∗∗∗^			0.08/86.49^∗∗∗^	0.08/86.49^∗∗∗^
SES	0.19^∗∗∗^	0.16^∗∗∗^			–0.16^∗∗∗^	–0.15^∗∗∗^		
Online hours per week	–0.07^∗∗^	–0.04			0.23^∗∗∗^	0.13^∗∗∗^		
2. *The Dark Triad and Internet use preference*			0.13/42.10^∗∗∗^	0.17/44.26^∗∗∗^			0.26/110.23^∗∗∗^	0.34/112.59^∗∗∗^
Machiavellianism		–0.07^∗∗^				0.18^∗∗∗^		
Psychopathy		–0.16^∗∗∗^				0.21^∗∗∗^		
Narcissism		0.02				0.06^∗∗^		
Information communication		0.02				0.05^∗^		
Recreation		−0.06^∗^				0.20^∗∗∗^		
Information acquisition		0.27^∗∗∗^				–0.15^∗∗∗^		
Online transaction		–0.08^∗∗^				0.14^∗∗∗^		

*^∗^*p* < 0.05, ^∗∗^*p* < 0.01, ^∗∗∗^*p* < 0.001.*

### The Mediation of Internet Use Preference on the Relationship Between the Dark Triad and Internet Adaptation

**FIGURE 1:**
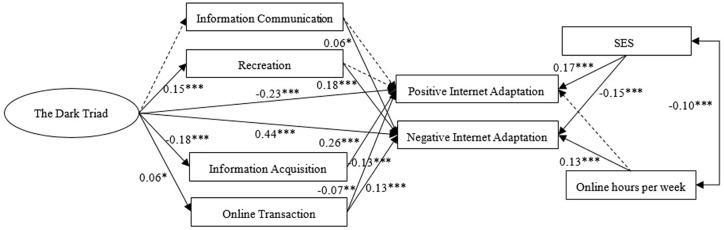
The mediation of Internet use preferences in the relation between the Dark Triad and Internet adaptation.

SEM was used to examine the relationship between the Dark Triad, Internet use preferences and Internet adaptation. The fit indices indicated a reasonable fit for the model: *X*^2^/*df* = 9.87; RMSEA = 0.068; NFI = 0.94; GFI = 0.98; CFI = 0.95; TLI = 0.89. Our analyses revealed that, when controlling for SES and online hours per week, Internet use preferences partially mediated the relationship between the Dark Triad and Internet adaptation, except that information communication and recreation did not mediate the relationship between the Dark Triad and positive Internet adaptation. In other words, recreation partially mediated the effect of the Dark Triad on negative Internet adaptation, and the mediation explained 5.8% of the total effect. Both information acquisition and online transaction partially mediated the effect of the Dark Triad on positive/negative Internet adaptation, and the mediation effects accounted for 16.9, 5.6, 1.8 and 1.7% of the total effects, respectively (see Figure 1).

## Discussion

### Correlation Among the Dark Triad, Internet Use Preferences and Internet Adaptation of Chinese Adolescents

Pearson correlation and regression analyses indicated that information acquisition, recreation and online transaction significantly predicted Internet adaptation. Therefore, hypothesis 1 was mainly supported except that the nature of the information communication was not clear. It might be because information communication covers a wide range of positive, neutral and negative information from eating and drinking to studying and using new Internet features.

The correlation and regression results also suggested that, in general, the Dark Triad negatively predicted positive Internet adaptation and positively predicted negative Internet adaptation. Compared with positive Internet adaptation, the three Dark Triad dimensions had more predictive power for negative Internet adaptation. Therefore, hypothesis 2 was mainly supported. The close correlation between the Dark Triad and negative Internet adaptation showed that adolescents who scored higher on the Dark Triad are more likely to develop negative adaptation behaviors, such as escaping from school using the Internet, avoiding relationships in reality, and Internet problem behaviors (Ang et al., 2010; Carpenter, 2012; Pabian et al., 2015).

The result also showed that psychopathy was a stronger predictor of Internet adaptation than Machiavellianism or narcissism. One possible reason is that individuals with high psychopathy are impulsive, lack of empathy and responsibility (Qin and Xu, 2013) and have negative social adaptation characteristics (Repacholi et al., 2003). Since Internet adaptation is a special kind of social adaptation, it should be highly correlated with psychopathy.

### The Mediation of Adolescents’ Internet Preferences on the Relationship Between the Dark Triad and Internet Adaptation

The result of SEM analysis revealed that hypothesis 3 was supported generally as Internet use preferences, except information communication, partially mediated the relationship between the Dark Triad and Internet adaptation. As mentioned above, information communication involved both positive and negative information. In the current measurement framework, it should be regarded as a neutral preference. Therefore, it was not a significant correlation of the relationship between the Dark Triad and Internet adaptation.

Since the Dark Triad traits were regarded as a fast or opportunistic life strategy, an impulsive behavioral style with low self-control (Figueredo et al., 2006; Jonason and Tost, 2010; Pabian et al., 2015), they could positively predict recreation and online transaction that involve relaxing from pressure and negatively predict information acquisition that involves insisting on learning. In short, teenagers with the Dark Triad are more likely to have recreation and online transaction preferences and less likely to have information acquisition preferences, which will further develop negative Internet adaptation. The relationships among the Dark Triad, Internet use preference and Internet adaptation can be explained by the uses and gratification theory and social network theory. According to this theory, personality traits (e.g., the Dark Triad traits) have an effect on individual needs and further determine Internet use preferences (Katz et al., 1973; Song et al., 2004). Social network theory also holds that personality affects motivation and behavior (e.g., Internet use preferences) in the process of social interaction, thus influencing social outcomes (e.g., Internet adaptation) (Amichai-Hamburger, 2002; Lei, 2016).

Meanwhile, the Dark Triad tends to develop directly negative Internet adaptation and not positive Internet adaptation. And further results suggested that the direct effect of the Dark Triad on Internet adaptation is more significant than the indirect (medicating) effect. This may suggest that in addition to Internet use preferences, there are other mediating variables (e.g., Internet use needs or motivations) that have not been discussed, and their existence weakens the mediating effect of Internet use preference. Furthermore, the differential effect of the Dark Triad and Internet use preferences on positive and negative adaptation also reflects that the opposite of negative Internet adaptation is not positive Internet adaptation (Zou et al., 2012). They may be two different variables.

### Limitations and Future Research

The present study focused on the Dark Triad, which has received less attention in Chinese culture, and examined the relations among the Dark Triad, Internet use preferences and Internet adaptation. Theoretically, it expanded the applicable environment of the Dark Triad, emphasized its effect on Internet use, and verified some theories of Internet use. In practice, the results indicated that attention should be paid to the combination of internal personality traits and explicit Internet use preferences in Internet adaptation interventions, and it should be noted that a decrease in negative Internet adaptation does not mean an increase in positive Internet adaptation. Improving positive Internet adaptation is a long-term process that needs to be monitored.

Moreover, there were some limitations in this study. First, the Dark Triad traits of adolescents are not very obvious due to the Dark Triad is a kind of socialized personality that changes with individual growth. The score of the Dark Triad traits were low in the adolescence (e.g., Compared with the total score of 7, the average score of Machiavellianism was only 1.91). This is also an important reason for the significant but weak correlation between the Dark Triad and Internet use preference and the weak partial mediation of Internet use preference. Therefore, we should pay more attention to the development of the Dark Triad from adolescence to adulthood.

Second, although the reliability and validity of the Chinese version of Dirty Dozen have been confirmed and the measurement of the Dark Triad of adolescents has been explored in previous research, the applicability of the Dark Dozen in adolescents still needs to be further discussed. For example, as subclinical traits, psychopathy, and narcissism are not very common in growing adolescents.

Third, as mentioned, a variety of motivations and needs may play crucial roles in the relationship between the Dark Triad and Internet use preference. Further research should explore the roles of these motivations and needs (such as social interaction motivation, relaxation motivation, etc.) in the relationship between personality and Internet behavior.

Finally, cross-sectional data were used in this study, which theoretically offered a possible mechanism of the relations among the Dark Triad, Internet use preferences and Internet adaptation. However, it is necessary to use longitudinal data to further explore the causal relations among these variables.

## Data Availability

The raw data supporting the conclusions of this manuscript will be made available by the authors, without undue reservation, to any qualified researcher.

## Ethics Statement

This study was carried out in accordance with the recommendations of the Ethics Review Committee of Beijing Forestry University with written informed consent from all subjects. All subjects gave written informed consent in accordance with the Declaration of Helsinki. Written informed consent was also obtained from the parents of all participants. The protocol was approved by the Ethics Review Committee of Beijing Forestry University.

## Author Contributions

CJ came up with the study concept. BW and AJ performed the investigation and statistical analysis. CJ wrote the first draft of the manuscript. CJ and BW improved and revised the manuscript. All authors approved the final version of the manuscript before submission.

## Conflict of Interest Statement

The authors declare that the research was conducted in the absence of any commercial or financial relationships that could be construed as a potential conflict of interest.

## References

[B1] Amichai-Hamburger Y. (2002). Internet and personality. *Comput. Human Behav.* 18 1–10. 10.1016/S0747-5632(01)00034-6

[B2] Ang R. P. Tan K. A. Talib M. A. (2010). Normative beliefs about aggression as a mediator of narcissistic exploitativeness and cyberbullying. *J. Interpers. Violence* 26 2619–2634. 10.1177/0886260510388286 21156699

[B3] Block J. (2010). The five-factor framing of personality and beyond: some ruminations. *Psychol. Inq.* 21 2–25. 10.1080/10478401003596626

[B4] Carpenter C. (2012). Narcissism on facebook: self-promotional and anti-social behavior. *Pers. Individ. Dif.* 52 482–486. 10.1016/j.paid.2011.11.011

[B5] Chen J. W. (2001). *Theoretical and Empirical Research on Adolescent Social Adaptation: Structure Mechanism and Function.* Doctoral dissertation, Southwest University, Chongqing.

[B6] Cheng J. W. Liu H. S. (2010). Developing questionnaire for internet use preference. *China J. Health Psychol.* 18 625–627. 10.13342/j.cnki.cjhp.2010.05.038

[B7] China Internet Network Information Center (2019). *The 43nd China Statistical Report on Internet Development.* Available at: http://www.cnnic.cn/hlwfzyj/hlwxzbg/hlwtjbg/201902/P020190318523029756345.pdf (accessed February 28, 2019).

[B8] Deng L. Y. Wu Y. X. Kong R. Fang X. Y. (2014). Interactive influence of impulsiveness and parent-adolescent communication on adolescents’ internet addiction. *Psychol. Dev. Educ.* 2 169–176. 10.16187/j.cnki.issn1001-4918.2014.02.001

[B9] Eksi F. (2012). Examination of narcissistic personality traits’ predicting level of internet addiction and cyber bullying through path analysis. *Educ. Sci.* 12 1694–1706.

[B10] Figueredo A. J. Vásquez G. Brumbach B. H. Schneider S. M. Sefcek J. A. Tal I. R. (2006). Consilience and life history theory: from genes to brain to reproductive strategy. *Dev. Rev.* 26 243–275. 10.1016/j.dr.2006.02.002

[B11] Figueredo A. J. Vásquez G. Brumbach B. H. Schneider S. M. R. (2007). The K-factor, covitality, and personality: a psychometric test of life history theory. *Hum. Nat.* 18 47–73. 10.1007/BF02820846 26181744

[B12] Geng Y. G. Sun Q. B. Huang J. Y. Zhu Y. Z. Han X. H. (2015). Dirty dozen and short dark triad: a Chinese validation of two brief measures of the dark triad. *Chin. J. Clin. Psychol.* 23 246–250. 10.16128/j.cnki.1005-3611.2015.02.013 24274044

[B13] Gosling S. D. Augustine A. Vazire S. E. A. (2011). Manifestations of personality in online social networks: self- reported face-book-related behaviors and observable profile information. *Cyberpsychology* 14 483–488. 10.1016/j.dr.2006.02.002 21254929 PMC3180765

[B14] He J. B. Zhu P. P. Nie Y. F. Ying S. Y. (2017). The influence of personality on internet addiction and its psychological mechanisms: a review. *Chin. J. Clin. Psychol.* 25 221–224. 10.16128/j.cnki.1005-3611.2017.02.006

[B15] Hughes D. J. Rowe M. Batey M. Lee A. (2012). A tale of two sites: twitter vs. facebook and the personality predictors of social media usage. *Comput. Human Behav.* 28 561–569. 10.1016/j.chb.2011.11.001

[B16] Ji A. T. (2018). *Adolescents’ Internet Adaptation: Structure, Characteristics and Influence Factors.* Master’s thesis, Beijing Forestry University, Beijing.

[B17] Jin C. C. (2013). *Social Adjustment of Juvenile Delinquents: The Characteristics and Factors.* Beijing: Central Compilation and Translation Press.

[B18] Jonason P. K. Kavanagh P. S. Webster G. D. Fitzgerald D. (2011). Comparing the measured and latent dark triad: are three measures better than one? *J. Methods Meas. Soc. Sci.* 2 28–44. 10.2458/azu_jmmss.v2i1.12363

[B19] Jonason P. K. Tost J. (2010). I just cannot control myself: the dark triad and self-control. *Pers. Individ. Dif.* 49 611–615. 10.1016/j.paid.2010.05.031

[B20] Katz E. Blumler J. G. Gurevitch M. (1973). Uses and gratifications research. *Public Opin. Q.* 37 509–523. 10.1086/268109

[B21] Kayis A. R. Satici S. A. Yilmaz M. F. (2016). Big five-personality trait and internet addiction: a meta-analytic review. *Comput. Human Behav.* 63 35–40. 10.1016/j.chb.2016.05.012

[B22] Kowalski R. M. Giumetti G. W. Schroeder A. N. Lattanner M. R. (2014). Bullying in the digital age: a critical review and meta-analysis of cyberbullying research among youth. *Psychol. Bull.* 140 1073. 10.1037/a0035618 24512111

[B23] Lee S. M. (2014). The relationships between higher order thinking skills, cognitive density, and social presence in online learning. *Internet High. Educ.* 21 41–52. 10.1016/j.iheduc.2013.12.002

[B24] Lei L. (2016). *Psychology of Internet: The Rise of New Psychological and Behavioral Studies.* Beijing: Beijing Normal University Press.

[B25] Liu S. J. Jin C. C. (2018). The relationship between college students’ mobile phone addiction and learning burnout: personality as a moderator. *Chin. J. Special Educ.* 86–91.

[B26] Luo F. S. Zhang S. M. Shen D. Luo K. (2011). College students’ internet deviant behaviors and personality characteristics and coping styles. *Chin. J. Clin. Psychol.* 19 492–493. 10.16128/j.cnki.1005-3611.2011.04.041

[B27] Masten A. S. Coatsworth J. D. Neemann J. Gest S. D. Garmezy N. (1995). The structure and coherence of competence from childhood through adolescence. *Child Dev.* 66 1635–1659. 10.1111/j.1467-8624.1995.tb00956.x 8556890

[B28] Pabian S. De Backer C. J. S. Vandebosch H. (2015). Dark triad personality traits and adolescent cyber-aggression. *Pers. Individ. Dif.* 75 41–46. 10.1016/j.paid.2014.11.015

[B29] Podsakoff P. M. MacKenzie S. B. Lee J. Y. Podsakoff N. P. (2003). Common method biases in behavioral research: a critical review of the literature and recommended remedies. *J. Appl. Psychol.* 88 879–903. 10.1037/0021-9010.88.5.879 14516251

[B30] Qin F. Xu F. (2013). Review on the studies of the dark triad. *Adv. Psychol. Sci.* 21 1248–1261. 10.3724/SP.J.1042.2013.01248

[B31] Repacholi B. Slaughter V. Pritchard M. Gibbs V. (2003). “Theory of mind, machiavellianism, and social functioning in childhood,” in *Individual Differences in Theory of Mind*, eds Slaughter B. Repacholi V. (New York, NY: Psychology Press), 67–97. 10.4324/9780203488508

[B32] Song I. Larose R. Eastin M. S. Lin C. A. (2004). internet gratifications and internet addiction: on the uses and abuses of new media. *CyberPsychol. Behav.* 7 384–394. 10.1089/cpb.2004.7.384 15331025

[B33] Stead R. Fekken G. C. Kay A. Mc Dermott K. (2012). Conceptualizing the dark triad of personality: links to social symptomatology. *Pers. Individ. Dif.* 53 1023–1028. 10.1016/j.paid.2012.07.021

[B34] Yang Y. P. Jin Y. (2007). The development of the middle school student’s social adaptation scale. *Psychol. Dev. Educ.* 23 108–114. 10.3969/j.issn.1001-4918.2007.04.019 24789702

[B35] Yao M. Z. He J. Ko D. M. Pang K. (2014). The influence of personality, parental behaviors, and self-esteem on internet addiction: a study of Chinese college students. *Cyberpsychol. Behav. Soc. Netw.* 17 104–110. 10.1089/cyber.2012.0710 24003966 PMC3924803

[B36] Zhang W. J. Zhang H. (2014). Dark Triad of personality: dual effects and mechanisms. *J. Beijing Norm. Univ.* 38–47.

[B37] Zhou H. Long L. R. (2004). Statistical remedies for common method biases. *Adv. Psychol. Sci.* 12 942–950. 10.3969/j.issn.1671-3710.2004.06.018

[B38] Zhou Q. Wang Y. Deng X. Eisenberg N. Wolchik S. A. Tein J. (2008). Relations of parenting and temperament to Chinese children’s experience of negative life events, coping efficacy, and externalizing problems. *Child Dev.* 79 493–513. 10.1111/j.1467-8624.2008.01139.x 18489409 PMC2762348

[B39] Zhou Z. K. Liu Q. X. (2016). Psychology of internet: reconstruction of behavior. *Soc. Sci. China Rev.* 55–67.

[B40] Zou H. Yu Y. B. Zhou H. Liu Y. (2012). Theoretical model construction and confirmation of middle school students’ social adjustment assessment. *J. Beijing Norm. Univ.* 1 65–72. 10.3969/j.issn.1002-0209.2012.01.008

